# Recent Tuberculosis Work

**Published:** 1919-12-06

**Authors:** 


### RECENT TUBERCULOSIS WORK.

### Scrofula and Hodgkin's Disease.

The differential diagnosis between these two affections is known to be in early stages anything but easy, even with the help of a careful blood count. And the problem is not simplified by the modern recognition of a third glandular enlargement, called *Lymphogranuloma tuberculosum*, first described by Sternberg. Here the lymphatic tissues are widely involved, mostly beginning in the cervical glands and proceeding to the spleen, liver, and bones. Microscopically there are seen mono- and polynuclear cells rich in protoplasm, occasionally Langhan's giant cells, with many eosinophiles. Recently animal experiment has resulted in tuberculous infection in a large majority of cases. Clinically the disease generally manifests itself first in the posterior triangle of the neck, and the glands, at first soft, tend to get harder. Separate groups of glands are likely to be collectively enlarged, as in the axilla. Chylous ascites may be present, after removal of which abdominal glands have been palpated. Also a radiograph may show enlargement of those in the mediastinum, which may give rise to pressure symptoms. A splenic tumour is demonstrable in the majority of instances, and sometimes enlargement of the liver; these are indented uneven swellings. An early symptom is increase of skin pigmentation, diffuse or spotty, and mostly in the neighbourhood of the glandular enlargements. Diagnostically a certain amount of pyrexia is helpful, which may produce sweating and rigors, with perhaps diarrhœa. There is a tendency to serious effusions into all the usual cavities. The diazo reaction is not always positive and the red-blood corpuscles seem normal. The course is generally acute; a fatal termination has been known to occur in eighteen days; but on an average the disease lasts from six months to two years. In inoculated animals it is much less severe. The causal micro-organism is supposed to be the human type of *Bacillus tuberculosis*.

### Tuberculosis in the Insane.

Whether those of unsound mind die of tubercle so often because of inherent vulnerability, or because of necessarily inadequate hygiene and treatment, is a perennial controversy, although the verdict, on the whole, inclines distinctly to the former alternative. Löw, writing in a psychiatrical journal, has found tuberculosis to be the cause of death much more often in dementia præcox than in epileptic insanity, G.P.I., maniacal depressive conditions, or senile dementia. He thinks that this speaks against environment as the cause of the tuberculosis, as does also the fact that the disease continues rife in modern asylums, built and run on satisfactory hygienic lines. The conditions of life are much the same too for dementia præcox patients as for the others, except that cases of G.P.I. of course have a much shorter stay in the asylum.

The difficulties of diagnosing tuberculosis in lunatics are confirmed; variations in weight, for instance, occur quite commonly in mental patients without special apparent cause. He recommends regular taking of the temperature as the best indication.

### Training and the Dispensary Nurse.

In the nurses' supplement to the Austrian tuberculosis dispensary journal, before mentioned in The Hospital, a legal opinion on the controversy as to fully and only partially-trained nurses, a controversy which is topical over here just now, is given in favour of the first, or "diplomaed" nurses. (Some English sanitary authorities are now awarding increase of pay to those nurses only who are qualified for admission to the ranks of the College of Nursing.) The author recommends raising of the standing of the certificated nurses by securing the adoption of some badge to show their qualification. The practice of nurses advertising in the daily press should cease and be replaced by registry offices dealing in the first place only with certificated nurses. Reforms in sickness and accident insurance of nurses are necessary, as also in the matter of pensions; the modern conception of workers' rights as represented by trustworthy persons chosen from certificated nurses acting advisorily, must be acted up to. On the other hand, nurses themselves should look upon their calling not as a mere means of livelihood, but should also have high ideals about it.

